# Reference ranges for foetal nasal bone length, prenasal thickness, and interocular distance at 18 to 24 weeks’ gestation in low-risk pregnancies

**DOI:** 10.1186/s12884-017-1602-3

**Published:** 2017-12-12

**Authors:** Ayşegül Altunkeser, M. Kazım Körez

**Affiliations:** 1 Department of Radiology, Konya Education and Research Hospital, Health Sciences University, Konya, Turkey; 2 Department of Statistics, Faculty of Science, Selcuk University, Konya, Turkey; 3 Radyoloji Bölümü Hacı Şaban Mah, Sağlık Bilimleri Üniversitesi, Konya Eğitim ve Araştırma Hastanesi, Meram Yeni Yol Caddesi, No: 97, PK Meram, Konya, 42090 Turkey

**Keywords:** 2D ultrasound, Foetal nasal bone length, Prenasal thickness, Interocular distance

## Abstract

**Background:**

The aim of the present study was to establish the normal ranges for foetal nasal bone length (NBL), prenasal skin thickness (PNT), interocular distance (IOD), and ratio of prenasal thickness to- nasal bone length (PNT/ NBL) at 18–24 weeks using two-dimensional (2D) ultrasound.

**Methods:**

This study was a retrospective study of prenatal ultrasonographic records from 407 foetuses between 18 and 24 weeks gestational age (GA). The NBL, PNT, IOD, PNT/ NBL ratio, biparietal diameter (BPD), and femur length (FL) were investigated. The relationships among NBL, PNT, IOD, PNT/ NBL, and GA were evaluated. Additionally, descriptive statistics for NBL, PNT, and IOD values for each gestational week were obtained.

**Results:**

There was a significant association between GA and NBL, PNT, and IOD between 18 and 24 weeks. NBL increased from a mean of 5.5 mm to 8.3 mm, PNT increased from a mean of 3.5 mm to 5.1 mm, and IOD increased from a mean of 11.1 mm to 14.5 mm. PNT/NBL ratio did not change with gestational age.

**Conclusions:**

This study showed normal ranges for NBL, PNT, IOD, and PNT/ NBL ratios for foetuses between 18 and 24 weeks in low-risk pregnancies. There was a positive linear relationship between GA and NBL, PNT, and IOD. The PNT/NBL ratio might be a more useful measurement than NBL or PNT alone.

**Electronic supplementary material:**

The online version of this article (10.1186/s12884-017-1602-3) contains supplementary material, which is available to authorized users.

## Background

Down syndrome is defined by specific set of facial properties comprising a flat facial profile, a small nose, ocular hypotelorism, and an excessive amount of skin. This syndrome was first reported by Langdon Down in 1866 [1]. Improvements in ultrasonography make evaluating these properties easier. Nasal bone and nuchal translucency measurements are markers used in the first trimester, while nasal bone length (NBL) and prenasal thickness (PNT) are among the proposed markers for Down syndrome in the second trimester [2, 3]. In the second trimester, the nasal bone measurement has to be performed at the exact midsagittal plane, with which the vomer is visualized [4, 5]. At the same time, it has also been reported that the measurements obtained from parasagittal and oblique planes do not reflect true values [4]. In this study, we aimed to obtain reference ranges for NBL, PNT, interocular distance (IOD), and the ratio between prenasal thickness and nasal bone length (PNT/NBL) in foetuses without known anomalies between 18 and 24 weeks’ gestation via two-dimensional (2D) ultrasound.

## Methods

Four hundred seven pregnant Turkish women in whom obstetric ultrasonography (US) was performed for anomaly screening in the radiology department of our hospital between November 2013 and May 2014 were included in this cross-sectional study. Foetuses with sonographically congenital anomalies were excluded. Additionally, pregnancies with other complications such as diabetes, chronic hypertension, early onset growth restriction, or HIV were excluded. The study was approved by the Ethics Committee of the Faculty of Medicine, Necmettin Erbakan University and was conducted in accordance with the Declaration of Helsinki. Ultrasonography was performed by one radiologist with at least 10 years of experience in obstetric US with 2D ultrasound devices (Acuson Antares; Siemens, Erlangen, Germany and Famio 8; Toshiba, Tokyo, Japan). Along with biparietal diameter and femur length, NBL, PNT, and IOD were obtained.

**Fig. 1:**
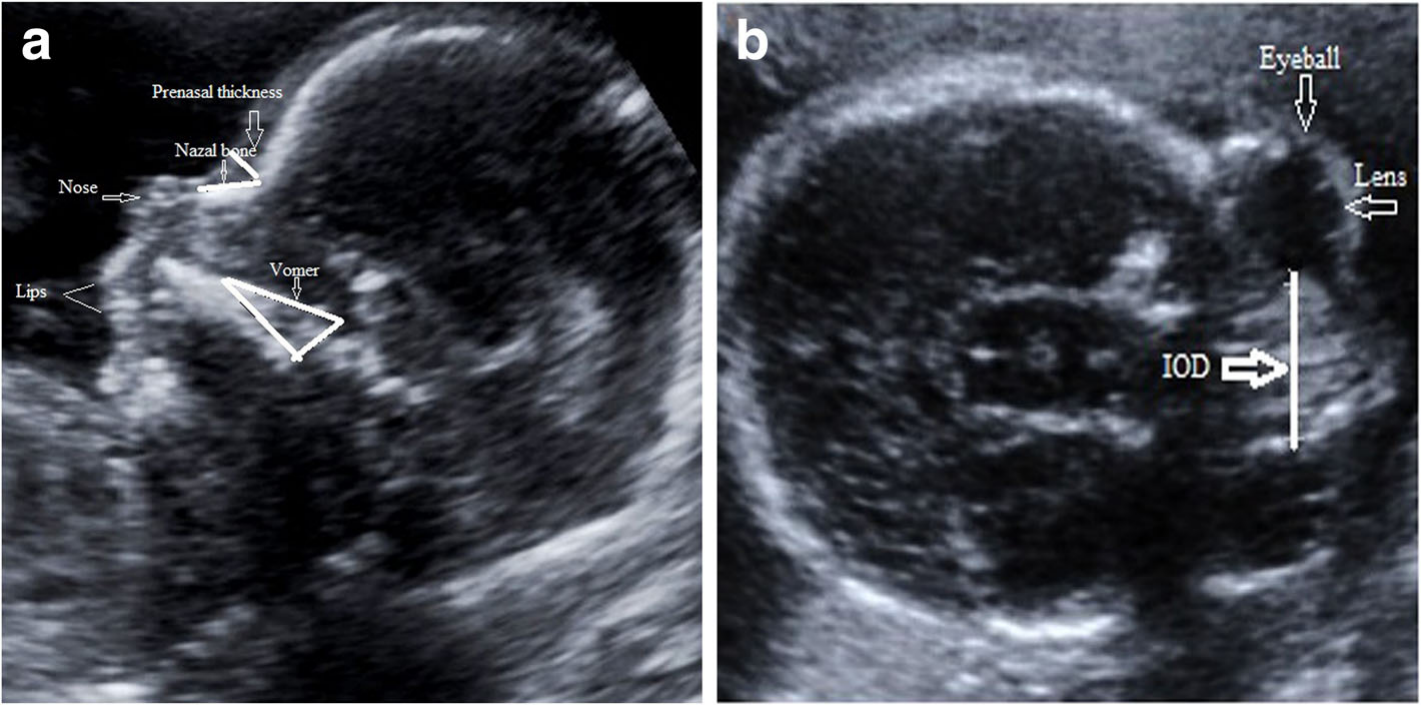
**a** Ultrasound image of a foetus showing the exact mid-sagittal plane of the face with its sonographic landmarks and measurement of nasal bone length and prenasal thickness. **b** The measurement of interocular distance in the axial plane. IOD: interocular distance

Measurements of NBL and PNT were performed at the midsagittal plane with 2D ultrasound. The exact midsagittal plane was determined by finding the nose, upper and lower lips, maxilla, and chin anteriorly, and the secondary palate with the overlying vomeral bone posteriorly [6]. The nasal bone was measured from the junction between the nasal and frontal bones to the distal edge of the white ossification line. The PNT was determined as the shortest distance between the anterior edge of the lowest segment of the frontal bone and the frontal skin [3]. The interocular distance was found as the distance between the inner borders of the orbits at the level at which eyeballs and lenses were symmetrical at the axial plane [6] (Fig. 1a and b).

Relationships between gestational age and NBL, PNT, IOD, and PNT/NBL ratio were investigated. Additionally, descriptive statistical analyses of NBL, PNT, and IOD for each gestational age (GA) were conducted.

### Statistical analysis

Multiple and simple linear regressions and Pearson’s correlations were used to determine the significance of the relationships between GA and NBL, PNT, IOD, or PNT/ NBL ratio. The Kolmogorov–Smirnov test was used to confirm the normality of their distributions. The arithmetic mean and standard deviation of each sample was calculated and statistically compared using ANOVA (F-test). Statistical analysis was performed using IBM SPSS 15.0, and a *p* < 0.05 was considered to be significant.

## Results

**Fig. 2:**
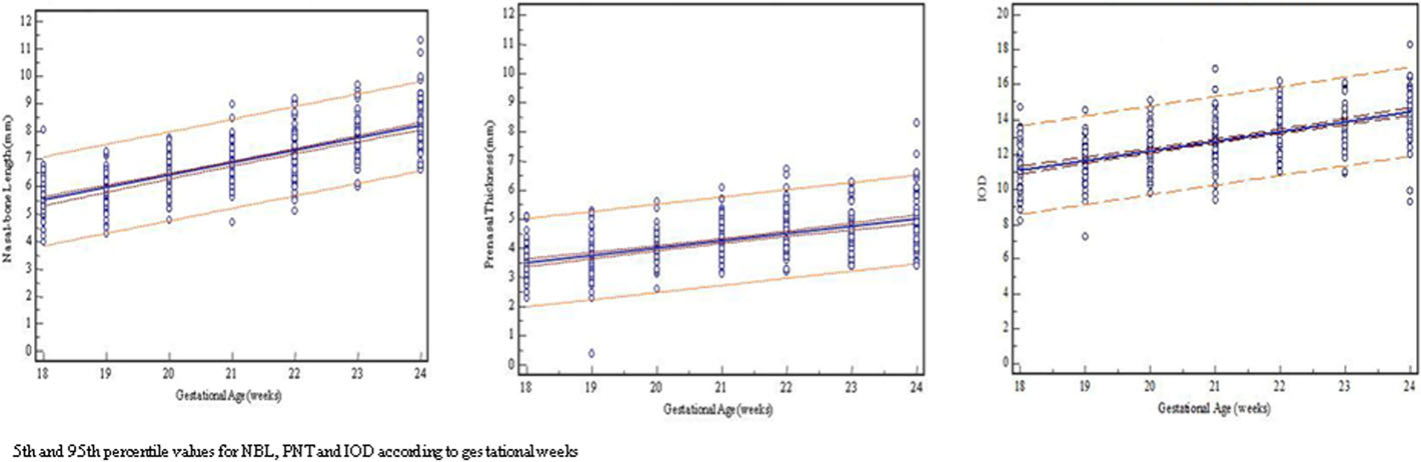
5th and 95th percentile values for NBL, PNT and IOD according to gestational weeks

The study group included 407 foetuses at 18 to 24 weeks’ gestation (mean, 21 weeks). The average patient age was 24 years. Only 377/407 NBL and PNT and 406/407 IOD measurements were successfully obtained because of inappropriate foetal position. For each GA, NBL, IOD, PNT and PNT/NBL ratio values were calculated (Table 1). Additionally, the percentile values were obtained for NBL, PNT, IOD and PNT/NBL ratio according to gestational week. Between 18 and 24 weeks, mean NBL increased from 5.5 mm to 8.3 mm, mean IOD increased from 11.1 mm to 14.5 mm, and mean PNT increased from 3.5 mm to 5.1 mm. PNT/NBL ratio did not change with GA (Table 2). Significant positive linear relationships were found between GA and NBL, IOD, and PNT, in said order (linear regression *p* < 0.001) and are represented in Fig. 2. The values for NBL, IOD, and PNT were estimated using regression equations:

**Table 1:** Results of NBL, PNT and IOD According to Gestational Weeks

Gestational Weeks	NBL		PNT		IOD	
	*n*	*Mean*	*n*	*Mean*	*n*	*Mean*
18	51	5.53	51	3.52	54	11.15
19	54	5.83	54	3.63	58	11.55
20	47	6.53	47	4.03	53	12.30
21	60	6.79	60	4.38	68	12.79
22	55	7.29	55	4.63	57	13.49
23	55	7.63	55	4.48	60	13.64
24	55	8.33	55	5.06	56	14.54

**Table 2:** Analysis Results of NBL, PNT and IOD According to Gestational Weeks

Variable	Gestational Age (weeks)							*p*
	18	19	20	21	22	23	24	
NBL	5.53 ± 0.73^a^	5.83 ± 0.66^a^	6.53 ± 0.74^b^	6.79 ± 0.76^b^	7.29 ± 0.94^c^	7.63 ± 0.79^c^	8.33 ± 1.02^d^	p < 0.001*
PNT	3.52 ± 0.70^a^	3.63 ± 0.82^ab^	4.03 ± 0.56^bc^	4.38 ± 0.69^cd^	4.63 ± 0.77^de^	4.48 ± 0.75^d^	5.06 ± 0.97^e^	p < 0.001*
IOD	11.15 ± 1.36^a^	11.55 ± 1.25^a^	12.30 ± 1.31^b^	12.79 ± 1.29^b^	13.49 ± 1.17^c^	13.64 ± 1.09^c^	14.54 ± 1.48^d^	p < 0.001*
PNT/NBL	0.63 ± 0.11	0.62 ± 0.14	0.62 ± 0.10	0.65 ± 0.11	0.64 ± 0.11	0.59 ± 0.11	0.61 ± 0.11	*0.141*

^a,b,c,d,e^indicate differences for each group. *indicates statistical significance between gestational weeks and the variable. *p < 0.05* for the differences between subgroups. Values are expressed as the mean ± SD

*NBL* Nasal bone length, *PNT* Prenasal thickness, *IOD* Interocular distance

NBL = −2.720 + 0.456(GA).

IOD = 1.134 + 0.554(GA).

PNT = −0.938 + 0.247(GA).

In which GA is gestational age. In all cases, p was <0.001, and the *R* ^2^ values were 0.55, 0.42, and 0.28, respectively (Additional file 1).

## Discussion

This study defines normal reference values for NBL and PNT obtained at the exact midsagittal plane, and IOD obtained in the axial plane of the foetal face at 18 to 24 weeks’ gestation by use of 2D ultrasound. Sonographic landmarks, including the nose, upper and lower lips, maxilla, and chin, are also visible in parasagittal and oblique sections of the foetal profiles. Persico et al. [4] compared NBLs taken in the parasagittal, oblique, and exact midsagittal planes using the multiplanar mode in 3D ultrasound. These researchers reported that the parasagittal and oblique scanning planes may produce different degrees of under- or over-estimation of the nasal bone length compared to the exact midsagittal plane, and the vomer is the only sonographic landmark for the midsagittal plane [4]. Nasal bone absence or hypoplasia is one of the most important markers of Down syndrome [7]. For this reason, the reliability and repeatability of NBL measurements are important. Additionally, the vomeral bone detected in the midsagittal plane can easily be seen in 2D ultrasonography. We attempted to display the vomer using 2D ultrasound, and we used the vomer as a marker for making accurate measurements.

The importance of the PNT as a marker for Down syndrome has increased in recent years. Maymon et al. [3, 8] reported that PNT thickness increased in foetuses with Down syndrome in the second trimester. In another study by Persico et al. [9], it was found that PNT alone could provide a highly sensitive means of screening for Down syndrome in the second trimester. The foetal profile is routinely examined in the second trimester screening, and the midsagittal plane can be used to assess both NBL and PNT. For this reason, it is practical to measure both of them.

In addition, the IOD can be helpful in defining anomalies involving the development of foetal orbits, which can be indicative of various anomalies and aneuploidy [10, 11]. Moreover, because PNT and IOD measurements are independent of the presence of the nasal bone, they can be suggested as two sonographic markers that could be combined in detecting affected foetuses.

Ethnicity reportedly affects nasal bone length [12, 13]. Carolyn et al. [14] noted that race and ethnicity significantly affected the mean regression line of the expected NBL among second trimester foetuses. However, Sonek et al. reported that NBL was not changed between African-American and Caucasian populations [15]. In our Turkish population, NBL values between 18 and 24 gestational weeks were compatible with those of Sonek’s population. Along with this finding, genetic sonographic norms are needed to obtain race- and ethnicity-specific formulas for NBL.

Gonzalez et al. [16] emphasized that the PNT/NBL ratio is a promising marker for sonographic screening for Down syndrome in low-risk populations. Szabo et al. also stressed that the ratio has high sensitivity and specificity [17]. In several studies, it was shown that the PNT/NBL ratio was stable and was 0.61 and 0.57 throughout gestation and the second trimester, respectively [3, 18]. Moreover, in another study, at 11–14 weeks of gestation it was reported that the ratio was 0.6 and are not altered by Crown-Rump Length [19]. We found that the PNT/NBL ratio did not change with gestational age. These findings are compatible with those of previous studies. Therefore, since the PNT/NBL ratio did not change with gestational week and was constant, we think that it might be practical to use in anomaly screening. Along with this finding, the prospective studies are needed to assess screening performance for the sonographic markers of NBL, PNT, and PNT/NBL ratio.

This study had several limitations and difficulties. The measurements were performed only by one examiner, so inter-observer variability could not be measured. Additionally, accurate NBL and PNT measurements in the proper plane are important, but they are difficult and time-consuming. We found the measurements could not be correctly obtained in 31 cases because of inappropriate foetal position. In addition, because of the absence of a foetus with Down Syndrome, we could not compare foetuses with and without Down Syndrome.

## Conclusions

This study provides the normal ranges for NBL, PNT, IOD, and PNT/NBL ratio at 18 to 24 weeks of pregnancy in low-risk cases in a Turkish population. In addition, we observed positive linear relationships between GA and NBL, PNT, and IOD but not the PNT/NBL ratio. The PNT/NBL ratio might be a more useful measurement than NBL or PNT alone, but this finding needs to be replicated in future studies. The emergence of noninvasive prenatal testing as a noninvasive accurate screening tool for Down syndrome will likely limit the importance of 2nd trimester sonographic markers but until this technology is widely available to all populations, improvement of Down syndrome detection via sonographic markers will still likely have a role.

## Additional file

Additional file 1: Percentile Values for NBL, PNT, IOD and PNT/NBL Ratio According to Gestational Weeks. (DOCX 12 kb)

## Abbreviations

2D: Two-dimensional
BPD: Biparietal diameter
FL: Femur length
GA: Gestational age
IOD: Interocular distance
NBL: Foetal nasal bone length
PNT: Prenasal skin thickness
US: Ultrasonography
