# Supplementary material for: Reference ranges for foetal nasal bone length, prenasal thickness, and interocular distance at 18 to 24 weeks’ gestation in low-risk pregnancies
Source: BMC Pregnancy Childbirth. 2017 Dec 12;17:416. doi: 10.1186/s12884-017-1602-3 (PMC5727986; doi:10.1186/s12884-017-1602-3)
Supplement: Additional file 1: — Percentile Values for NBL, PNT, IOD and PNT/NBL Ratio According to Gestational Weeks. (DOCX 12 kb) [file 12884_2017_1602_MOESM1_ESM.docx]

**Table 3**: Percentile Values for NBL, PNT, IOD and PNT/NBL Ratio According to Gestational Weeks

|  | NBL | | | | PNT | | | | IOD | | | | PNT/NBL | | | |
| --- | --- | --- | --- | --- | --- | --- | --- | --- | --- | --- | --- | --- | --- | --- | --- | --- |
| Week | 5P | 25P | 75P | 95P | 5P | 25P | 75P | 95P | 5P | 25P | 75P | 95P | 5P | 25P | 75P | 95P |
| 18 | 4.40 | 5.10 | 5.87 | 6.79 | 2.50 | 3.02 | 3.90 | 5.09 | 9.22 | 10.00 | 12.00 | 13.46 | 0.48 | 0.57 | 0.71 | 0.83 |
| 19 | 4.64 | 5.40 | 6.20 | 7.04 | 2.54 | 3.10 | 4.00 | 5.08 | 9.60 | 10.70 | 12.30 | 13.42 | 0.48 | 0.54 | 0.70 | 0.89 |
| 20 | 5.20 | 6.12 | 7.20 | 7.53 | 3.18 | 3.70 | 4.40 | 4.97 | 10.23 | 11.20 | 13.37 | 14.25 | 0.46 | 0.55 | 0.70 | 0.76 |
| 21 | 5.75 | 6.25 | 7.35 | 7.95 | 3.40 | 3.85 | 4.80 | 5.70 | 10.20 | 12.25 | 13.40 | 14.90 | 0.49 | 0.59 | 0.71 | 0.89 |
| 22 | 5.65 | 6.52 | 7.97 | 8.92 | 3.37 | 4.02 | 5.07 | 6.02 | 11.43 | 12.75 | 14.22 | 15.59 | 0.47 | 0.58 | 0.70 | 0.83 |
| 23 | 6.60 | 7.02 | 8.10 | 9.27 | 3.42 | 4.00 | 4.97 | 5.97 | 11.90 | 13.05 | 14.25 | 15.65 | 0.43 | 0.52 | 0.65 | 0.78 |
| 24 | 6.72 | 7.60 | 9.10 | 9.97 | 3.65 | 4.35 | 5.80 | 6.57 | 12.12 | 13.85 | 15.45 | 16.47 | 0.43 | 0.54 | 0.68 | 0.80 |

NBL: Nasal bone length

PNT: Prenasal thickness

IOD: Interocular distance
